# Biophysics in the Czech Republic: 70th anniversary of the institute of biophysics

**DOI:** 10.1007/s00249-025-01795-3

**Published:** 2025-09-28

**Authors:** Eva Bártová, Stanislav Kozubek, Aleš Kovařík

**Affiliations:** Department of Cell Biology and Epigenetics, Institute of Biophysics of the Czech Academy of Sciences, Královopolská 135, 612 00 Brno, Czech Republic

**Keywords:** Biophysics, Biophysical methods, Ionizing radiation, Radiotherapy, DNA damage repair

## Abstract

Biophysics is an interdisciplinary science that applies the theories and methods of physics to understand biological systems. It encompasses a wide range of topics, from the molecular mechanisms within cells to the physical properties of organisms and ecosystems. The goal of biophysics is to uncover the physical principles underlying the structure and function of biological molecules, cells, and cellular systems, providing a deeper understanding of life itself. The Institute of Biophysics, Czech Academy of Sciences (IBP) stands as a beacon of excellence in the field of biophysical research in the Czech Republic. This article delves into its history, structure, research areas, and major scientific achievements, highlighting the role of IBP in the global scientific community.

**Supplementary Information:**

The online version contains supplementary material available at 10.1007/s00249-025-01795-3.

## Biophysics: basic principles

Biophysics has its roots in the early twentieth century, with significant contributions from physicists and biologists who sought to explain biological phenomena using physical principles. The key milestone in the development of biophysics included the discovery of radiation (Becquerel et al. 1900; Röntgen 1895), which started an investigation of its biological effects and even the use of radiation in medicine for diagnostics and radiation treatment of cancer (Kustner 1923). Later on, in the fifties, a double helix structure of DNA was discovered by Watson and Crick (1953) and is considered a milestone in biology. Their model of the DNA double helix was based on the biophysical X-ray diffraction experiments of Rosalind Franklin (Franklin and Gosling 1953) reviewed by Cobb and Comfort (2023). Also, the development of techniques such as nuclear magnetic resonance (NMR) and cryo-electron microscopy (cryo-EM) for studying the structures of biological molecules was also considered a revolutionary biophysical approach (Bloch et al. 1946; Dubochet and Mcdowall 1981; Rabi 1937).

For decades, biophysical studies have taken many forms, including molecular biophysics, cellular biophysics, and system or medical biophysics. For example, molecular biophysics focuses on the physical properties and behaviors of biological molecules, such as proteins, nucleic acids, lipids, and carbohydrates. Important areas of study include protein folding, enzyme kinetics, and DNA/RNA dynamics (Bushhouse et al. 2022; Mlynsky et al. 2022; Stadlbauer et al. 2013). All these aspects are also addressed by cellular biophysics, solving the physical processes within and between cells, including their membrane fluidity, phase transitions, permeability, and role in signal transduction. Also, studies on cell signaling could use some biophysical approaches, including the use of a technique called Fluorescence Recovery After Photobleaching (FRAP) that enables the study of protein diffusion inside the cells (Lippincott-Schwartz et al. 2018; Stixová et al. 2011).

Also, many biophysicists are interested in systems biophysics that integrate multiple levels of biological organization, from molecules to ecosystems, to understand complex biological phenomena. For example, neurobiophysicists study the physical principles underlying the functioning of the nervous system; also, other medical biophysicists perform biomechanical studies exploring the mechanical properties of tissues and organs and study how physical factors contribute to physiological functions, which disorders lead to pathophysiological states in humans. Worth mentioning is also biophysical ecology or environmental biophysics leading to an understanding of the interactions between organisms and their environment (Campbell and Norman 1998; Monteith and Unsworth 2013).

## The Institute of Biophysics of the Czech Academy of Science: 70th anniversary

In the Czech Republic, the first established biophysical institute was the Institute of Biophysics of the Czech Academy of Sciences (Supplementary Figs 1 and 2). In January 1955, this institute arose from the Biophysical Laboratory of the Faculty of Medicine, a former University of J. E. Purkinje in Brno, now Masaryk University in Brno. The initial mission of the Institute was to research the effects of different types of radiation on various biological systems. Nowadays, researchers study the structure, function, and dynamics of biological systems (biomolecules, cell components, cells, and cell populations) using biophysical methods, including fluorescent protein technologies, CD spectroscopy, Raman spectroscopy, computational simulations, or electrochemistry of nucleic acids. The focus of interest is the investigation of the physical properties of nucleic acids.

Worth mentioning is also the fact that the history of biophysics in the former Czechoslovakia is closely connected with two outstanding scientists working at Masaryk University Brno in the early 1930s, the biophysicists Ferdinand Herčík and Vilém Laufberger. While Prof. Laufberger moved his scientific activities to Charles University in Prague in 1935, Ferdinand Herčík stayed in Brno and contributed to the recognition of biophysics as a field of science. Also, he initiated the construction of a new building of the Institute of Biophysics in Brno. Prof. Herčík’s scientific interests were diverse, ranging from radiobiology to molecular biophysics. These interests led him to initiate the establishment of a specialized laboratory dedicated to research in biophysics and especially biophysical methods. In 1927, Prof. Herčík published his first independent publication on biophysical research in plant models (Hercík 1927). In his scientific career, Prof. Herčík deepened his knowledge of biophysics during visits to the Lecomte du Noüy’s Department of Molecular Biophysics at the Pasteur Institute in Paris (1928, 1930–1931). Later, he moved to the Rockefeller Institute in New York, working in the biophysical laboratory of R.W.G. Wyckoff (1935–1936). Prof. Herčík studied the biological effects of all kinds of shortwave radiation, especially penetrating electromagnetic and corpuscular radiation (namely α-rays), on living organisms and their cells. The evidence for intervention theory that he obtained gradually led him to the theory of quantum biology. He became a pioneer of Czechoslovak biophysics and the founder of Czechoslovak radiobiology and radiotherapy. In 1936, Prof. Herčík was appointed full professor of general biology. In the meantime, he was employed at the Provincial Radiotherapy Institute (today’s Masaryk Memorial Cancer Institute) in Brno, where he devoted himself to radiotherapy and continued his experimental work. From 1947, he also devoted himself to electron microscopy of bacteriophages, which opened the sphere of molecular biology to Czechoslovak science (Hercik 1964). Among others, he was elected and appointed to many positions in institutions and societies, both domestic and foreign, e.g., from 1956, he was a member and subsequently the chairman of the United Nations Scientific Committee dealing with the effects of atomic radiation. The main office of this institution was in New York. From 1958, he was successively an expert and vice-chairman of the Board of Governors of the International Atomic Energy Agency in Vienna, an expert for radiobiology for UNESCO in Paris, and a member of the World Health Organization in Geneva. From 1963, he was a member of the committee of the International Association for Radiation Research.

In the early seventies, it is worth mentioning the active participation of researchers in international programs such as COMECON and Intercosmos, aimed at crewed and uncrewed space missions and astrophysics. Specifically, Dr. Antonín Vacek closely collaborated with the first Czechoslovak astronaut, Vladimír Remek (https://www.britannica.com/biography/Vladimir-Remek). In recent times, the important is also the fact that the two members of the European Molecular Biology Organization (EMBO) are permanently working at the Institute of Biophysics. Prof. Jiří Fajkus is interested in the genomics and proteomics of plants, focusing on the structure and function of plant telomeres. The second EMBO member is the current director of the IBP, Prof. Eva Bártová, working in the field of epigenetics and epitranscriptomics. She is mainly interested in the epigenetics of DNA damage response. Recently her team discovered that specific post-transcription modifications of RNA contribute to the repair process in the irradiated human genome (Kovaříková et al. 2023, 2020; Legartová et al. 2022).

## Major scientific achievements throughout the institute’s 70-year history

**Fig. 1:**
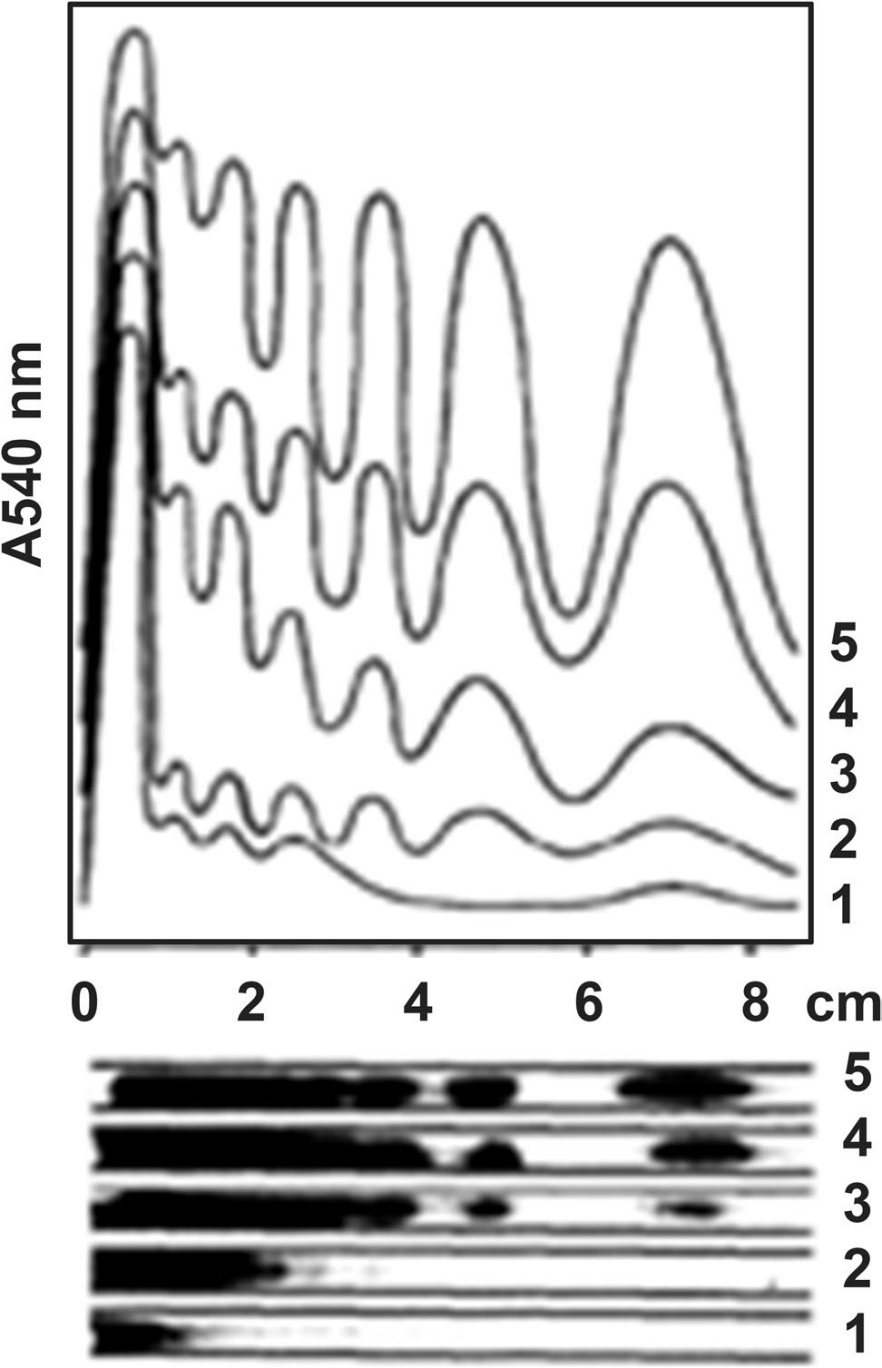
Electrophoretic separation of DNA isolated from thymus nuclei of normal mice (lane I) and from thymus nuclei of mice exposed to irradiation at 600 R (lanes 2–5). The chromatin was isolated 1, 2, 4, and 6 h (lanes 2–5) after the irradiation. Reproduced from Skalka et al. (1976) with permission of Elsevier

This article aims to summarize the most important scientific achievements of the IBP scientists since the Institute was established in 1955. In the fifties, global research trends in biophysics have largely been influenced by the discovery of a double helix structure of DNA (Watson and Crick 1953). The researchers of the newly formed institute have soon become aware of these breakthrough achievements. For example, cellular DNA content has been measured in plant cells by Dr. Vladimír Drášil and Dr. Milan Nermut (Nermut and Drasil 1958). A rather unfortunate period of the Cold War in the fifties stimulated research to study the impacts of radiation on living matters. Many researchers have, therefore, focused on radiation biology, studying the effect of ionizing radiation at the molecular level (Drasil and Soska 1958; Karpfel et al. 1959a, b; Soska et al. 1958). Dr. Jana Šlotová (director of the IBP in years 1998–2005) and colleagues investigated repair processes in genomes of irradiated plants (Šlotová et al. 1971). Dr. Miloslav Skalka and colleagues analyzed chromatin in the lymphoid tissues of mice exposed to gamma irradiation (Skalka et al. 1976). Their work provided evidence that chromatin is fragmented into nucleosomal units in irradiated cells (Fig. 1). They called the process enzymatic degradation of chromatin, later becoming known as programmed cell death. For the separation of DNA fragments, they used polyacrylamide gel electrophoresis, which was a revolutionary technique at those times (Fig. 1). Dr. Skalka later got a position at the International Atomic Energy Agency in Vienna, Austria.

**Fig. 2:**
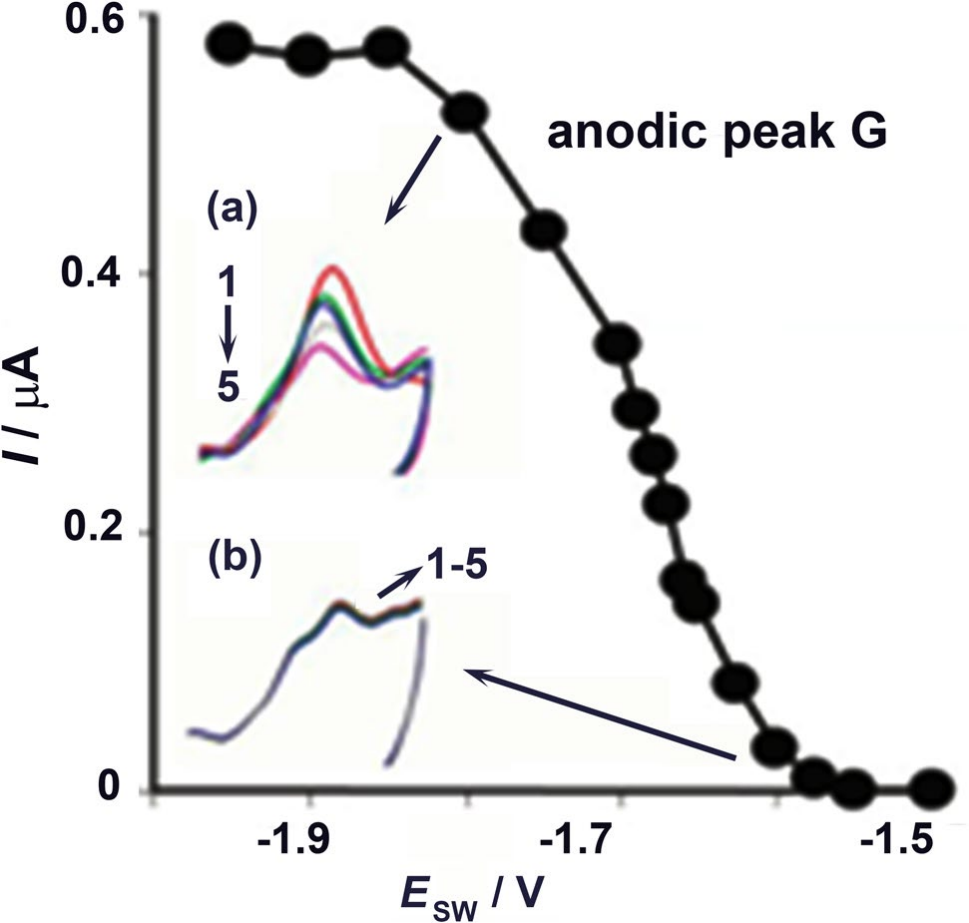
Cyclic voltammetry of DNA at HMDE. Dependence of anodic peak G (around − 0.3 V) on switching potential, E SW. Adapted from Jelen and Palecek (1986) with permission

In the late 1950s and early 1960s, Prof. Emil Paleček, an outstanding scientist, began investigating the electrochemical properties of nucleic acids and polysaccharides. His pioneering research demonstrated that DNA is electrochemically active and that its structure can be studied using biophysical techniques such as polarography (Paleček 1960). This important analytical method was invented by a Czech scientist, Prof. Jaroslav Heyrovský, who was awarded a Nobel Prize for chemistry in 1959. Electrochemistry of biomacromolecules, besides nucleic acids, (Jelen and Palecek 1986; Palecek and Bartosík 2012) (Fig. 2), proteins, polysaccharides, and glycoproteins (Palecek et al. 2015) became a key research direction of the IBP for more than six decades, mainly due to contributions from Emil Palecek during his long and productive life. Also, Prof. Viktor Brabec introduced carbon electrodes into the electrochemical NA research, showing that conformation states of DNA can be investigated using electrochemical approaches (Brabec and Palecek 1976). Prof. Viktor Brabec continued his research in the Institute in the field of molecular biophysics studying the effects of platinum compounds on DNA in the context of pharmacological research (Brabec and Kasparkova 2005). In the field of electrochemistry, Dr. Miroslav Fojta has been involved in the development of electrochemical biosensors for DNA damage (Fojta 2002) and in the development of techniques for redox DNA labeling (Hocek and Fojta 2011).

**Fig. 3:**
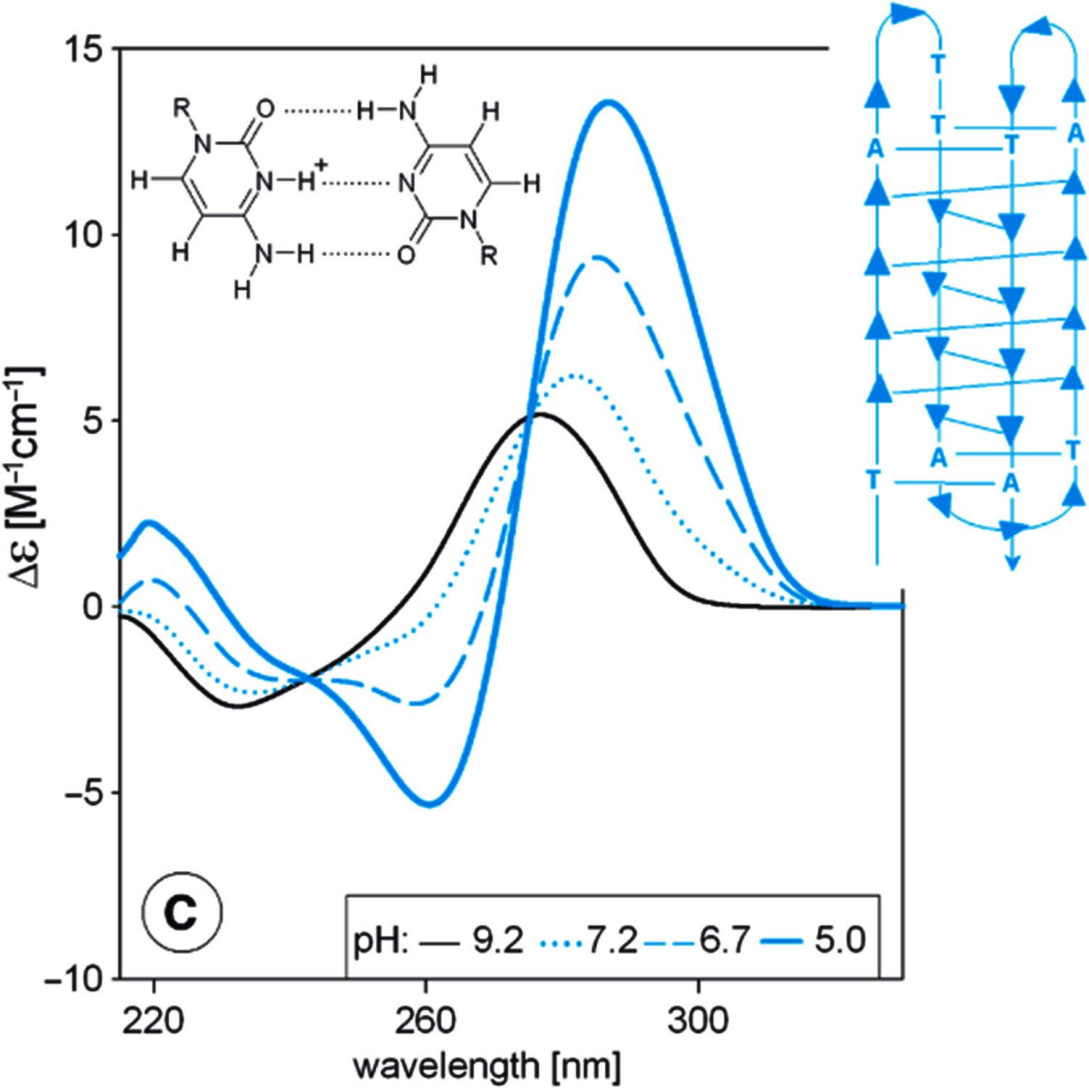
Example of CD spectra reflecting the acid-induced transition of a DNA fragment of a c-myc human oncogene into an intercalated cytosine quadruplex. Reproduced from Kypr et al. (2009) with permission

In the eighties, biophysical investigations of nucleic acids benefited from the advent of new spectroscopic methods. Specifically, groups of prof. Michaela Vorlíčková and Dr. Jaroslav Kypr applied, first in the Czech Republic, the circular dichroism method for studies of DNA structure (Vorlickova et al. 1980). Circular dichroism (CD) is a phenomenon originating from interactions of chiral molecules with circularly polarized electromagnetic rays. In the case of nucleic acids, the CD phenomenon originates because of folding absorbing nucleic acid bases into asymmetric helical arrangements. Utilizing CD, the authors characterized a wide family of B forms, A forms of DNA and RNA, left-handed Z forms, guanine quadruplexes, cytosine tetra-stranded i-motifs, and other non-canonical structures (e.g., Kypr et al. 2009) (Fig. 3). Further, Prof. Vorlíčková’s group discovered conformational properties of DNA fragments containing trinucleotide repeats (GCC)n and (GGC)n, whose expansion is correlated with the fragile X chromosome syndrome, a severe genetically determined disease (Fojtík et al. 2004; Fojtík and Vorlíčková 2001).

In the nineties, researchers realized that the DNA and chromatin structures needed to be viewed in a cellular context and not just as isolated molecules. At those times, the field of cell biology was rapidly evolving owing to technological progress in microscopic techniques allowing visualization of subcellular objects at a high resolution and with unprecedented specificity. For instance, Assoc. Prof. Stanislav Kozubek (director of the IBP in years 2009–2016) and Dr. Emílie Lukášová and colleagues exploited fluorescent in situ hybridization (FISH) to study chromosome and gene rearrangement in interphase nuclei exposed to γ-radiation (Kozubek et al. 1999). Their experiments suggested that intergenomic translocations occurring in malignant cells might be influenced by the positions of genes in the 3D-visualized cell nucleus. The topology of the cell nucleus and molecular composition of heterochromatin and euchromatin have attracted the attention of other young researchers. Using advanced microscopic methods, in combination with immunostaining of histones with specific antibodies, researchers found that epigenetic states of chromatin strongly influence DNA repair processes (Bártová et al. 2008) and can be modulated by histone deacetylation drugs (Bártová et al. 2008, 2011). These works were influential in our understanding of cellular responses to irradiation. The field of epigenetics also flourished in plant research, where epigenetic phenomena are particularly significant. Dr. Milan Bezděk (director of the IBP in the years 1990–1997) and Prof. Boris Vyskot became interested in cytosine methylation, which appears to be the most important epigenetic mark in DNA. Through fine genetic experiments using a demethylation drug, Dr. Janoušek and Prof. Vyskot discovered that sex determination in plants can be influenced by DNA methylation (Janousek et al. 1996). Further, Dr. Aleš Kovařík’s group investigated plant heterochromatin, showing that DNA methylation changes may occur in plants in response to environmental stress, such as drought and salinity (Kovarik et al. 1997). Advances in epigenetic research required the introduction of new methods, which have been elaborated for whole-genomic analyses of DNA methylation (Fulnecek and Kovarik 2014).

**Fig. 4:**
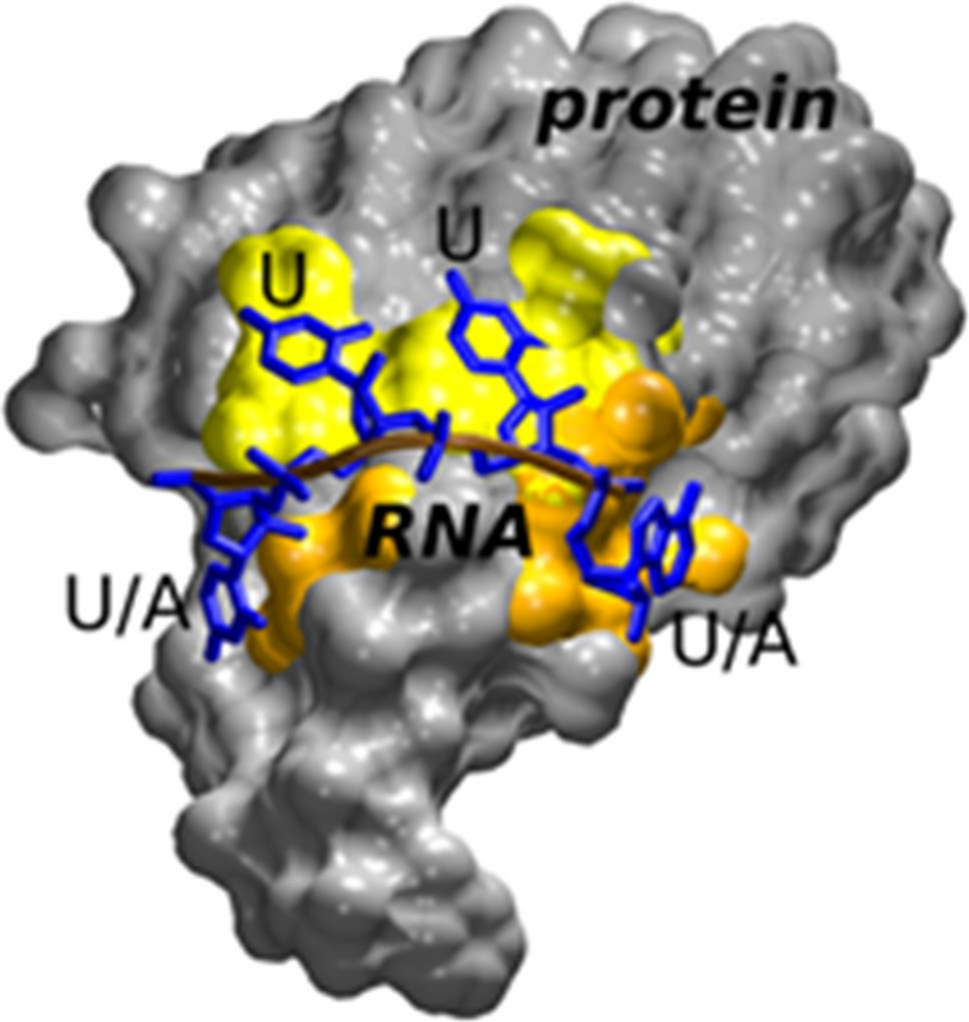
Example of molecular dynamics simulations of HuR protein critical for regulation of cellular mRNA stability. The RNA recognition domain (grey) contains classical RNA (blue) binding sites recognizing only uracil (yellow) and further unique binding sites capable of accepting either uracil or adenine (orange). Reproduced from Ripin et al. (2019) with permission

Towards the end of the millennium, biological research started to be influenced by computer science. Fast processors and more powerful algorithms allowed calculations of simple biological structures *ad initio*. Rapid technological progress has fascinated Prof. Jiří Šponer, who developed the field of molecular dynamics, allowing computer simulation of macromolecule processes (Sponer et al. 1996). He realized that more challenging than simply maintaining canonical helical structure is achieving a balanced description of the various non-canonical and/or unfolded nucleic acid structures, which are especially important in analyses of RNA functions, catalysis, dynamics, and drug targeting (Zgarbová et al. 2011; Ripin et al. 2019) (Fig. 4). Interactions of proteins with unusual DNA and RNA structures, including quadruplexes, have been studied by Prof. Vaclav Brázda (Brázda et al. 2014) and Dr. Michal Štros (Stros 2010). The discovery of small RNA molecules and their regulatory role in processes of differentiation and human pathology motivated Dr. Karel Souček and his colleagues, who noted that one of these RNA molecules, called miR-34a, plays a critical role in specific cancer progression (Slabáková et al. 2017).

Currently, we aim to constantly introduce technical innovations such as super*-*resolution microscopy, single-cell analyses, advanced in vitro and in vivo models, or genome editing to keep our research modern, efficient, and attractive. We increase the level of knowledge and education, the development of biotechnologies, and the transfer of research results to practical applications, particularly in the fields of diagnostics and treatment of deleterious human diseases and modern agriculture.

## Supplementary Information

Below is the link to the electronic supplementary material.

Supplementary file1 (TIF 805 KB)

Supplementary file2 (TIF 2611 KB)

## Data Availability

No additinal data and support is provided.

## References

[CR1] Bártová E, Krejcí J, Harnicarová A, Galiová G, Kozubek S (2008) Histone modifications and nuclear architecture: a review. J Histochem Cytochem 56:711–721 18474937 10.1369/jhc.2008.951251 PMC2443610

[CR2] Bártová E, Sustácková G, Stixová L, Kozubek S, Legartová S, Foltánková V (2011) Recruitment of Oct4 protein to UV-damaged chromatin in embryonic stem cells. PLoS ONE 6(12):e27281 22164208 10.1371/journal.pone.0027281 PMC3229488

[CR3] Becquerel H, Curie P, Curie F (1900) The uranium rays and the different physical properties of radiation coming from radioactive bodies. The new radioactive substances and the radiation emitted from these substances. Phys Z 2:563–564

[CR4] Bloch F, Hansen WW, Packard M (1946) Nuclear induction. Phys Rev 69:127–127

[CR5] Brabec V, Kasparkova J (2005) Modifications of DNA by platinum complexes—relation to resistance of tumors to platinum antitumor drugs. Drug Resist Updat 8:131–146 15894512 10.1016/j.drup.2005.04.006

[CR6] Brabec V, Palecek E (1976) Interaction of nucleic-acids with electrically charged surfaces. 2. Conformational-changes in double-helical polynucleotides. Biophys Chem 4:79–92 942864 10.1016/0301-4622(76)80009-9

[CR7] Brázda V, Hároníková L, Liao JCC, Fojta M (2014) DNA and RNA quadruplex-binding proteins. Int J Mol Sci 15:17493–17517 25268620 10.3390/ijms151017493 PMC4227175

[CR8] Bushhouse DZ, Choi EK, Hertz LM, Lucks JB (2022) How does RNA fold dynamically? J Mol Biol. 10.1016/j.jmb.2022.167665 35659535 10.1016/j.jmb.2022.167665 PMC9474645

[CR9] Campbell GS, Norman JM (1998) An introduction to environmental biophysics, 2nd edn. Springer-Verlag, New York, USA

[CR10] Cobb M, Comfort N (2023) What Rosalind Franklin truly contributed to the discovery of DNA’s structure. Nature 616:657–660 37100935 10.1038/d41586-023-01313-5

[CR11] Drasil V, Soska J (1958) Induced recovery of DNA synthesis in bone marrow from irradiated Guinea pigs. Biochim Biophys Acta 28:667–668 13560438 10.1016/0006-3002(58)90550-x

[CR12] Dubochet J, Mcdowall AW (1981) Vitrification of pure water for electron-microscopy. J Microsc-Oxford 124:Rp3–Rp4

[CR13] Fojta M (2002) Electrochemical sensors for DNA interactions and damage. Electroanal 14:1449–1463

[CR14] Fojtík P, Vorlícková M (2001) The fragile X chromosome (GCC) repeat folds into a DNA tetraplex at neutral pH. Nucleic Acids Res 29:4684–4690 11713318 10.1093/nar/29.22.4684 PMC92515

[CR15] Fojtík P, Kejnovská I, Vorlícková M (2004) The guanine-rich fragile X chromosome repeats are reluctant to form tetraplexes. Nucleic Acids Res 32:298–306 14718550 10.1093/nar/gkh179 PMC373289

[CR16] Franklin RE, Gosling RG (1953) Evidence for 2-chain helix in crystalline structure of sodium deoxyribonucleate. Nature 172:156–157 13072614 10.1038/172156a0

[CR17] Fulnecek J, Kovarik A (2014) How to interpret methylation sensitive amplified polymorphism (MSAP) profiles? BMC Genet 15:2 24393618 10.1186/1471-2156-15-2 PMC3890580

[CR18] Hercík F (1927) The photocapillary reaction of plant sap. Biochem J 21:1253–1258 16743956 10.1042/bj0211253 PMC1252050

[CR19] Hercik F (1964) Effect of cysteine + glycerol on capacity of irradiated cells of *Escherichia coli* B for phage T3. Folia Biol-Prague 10:307 14189808

[CR20] Hocek M, Fojta M (2011) Nucleobase modification as redox DNA labelling for electrochemical detection. Chem Soc Rev 40:5802–5814 21625726 10.1039/c1cs15049a

[CR21] Janousek B, Siroky J, Vyskot B (1996) Epigenetic control of sexual phenotype in a dioecious plant, *Melandrium album*. Mol Gen Genet 250:483–490 8602166 10.1007/BF02174037

[CR22] Jelen F, Palecek E (1986) Chemically reversible electroreduction of guanine in a polynucleotide chain. Biophys Chem 24:285–290 3768472 10.1016/0301-4622(86)85033-5

[CR23] Karpfel Z, Soska J, Drasil V (1959a) The effect of certain nucleotides and nucleosides on the regeneration of haemopoietic tissue after irradiation. Biofizika [Transl] 4:64–71 13628771

[CR24] Karpfel Z, Soska J, Drasil V (1959b) Effect of pyrimidine deoxyribonucleotides on the regeneration of bone-marrow in irradiated mice. Nature 183:1600–1601 13666834 10.1038/1831600b0

[CR25] Kovařík A, Koukalová B, Bezděk M, Opatrný Z (1997) Hypermethylation of tobacco heterochromatic loci in response to osmotic stress. Theor Appl Genet 95:301–306

[CR26] Kovaříková AS, Štixová L, Kovarík A, Komurková D, Legartová S, Fagherazzi P, Bártová E (2020) N-Adenosine methylation in RNA and a reduced mG/TMG level in non-coding RNAs appear at microirradiation-induced DNA lesions. Cells-Basel 9(2):360 10.3390/cells9020360 PMC7072662 32033081

[CR27] Kovaříková AS, Štixová L, Kovarík A, Bártová E (2023) PARP-dependent and NAT10-independent acetylation of N4-cytidine in RNA appears in UV-damaged chromatin. Epigenet Chromatin 16(1):26 10.1186/s13072-023-00501-x PMC10268562 37322549

[CR28] Kozubek S, Lukásová E, Marecková A, Skalníková M, Kozubek M, Bártová E, Kroha V, Krahulcová E, Slotová J (1999) The topological organization of chromosomes 9 and 22 in cell nuclei has a determinative role in the induction of t(9,22) translocations and in the pathogenesis of t(9,22) leukemias. Chromosoma 108:426–435 10654081 10.1007/s004120050394

[CR29] Kustner H (1923) Scattered radiation in the diagnostic and therapeutic implementation of X-ray diffraction. Naturwissenschaften 11:97–106

[CR30] Kypr J, Kejnovská I, Renciuk D, Vorlícková M (2009) Circular dichroism and conformational polymorphism of DNA. Nucleic Acids Res 37:1713–1725 19190094 10.1093/nar/gkp026 PMC2665218

[CR31] Legartová S, Kovaříková AS, Suchánková JB, Polásek-Sedlácková H, Bártová E (2022) Early recruitment of PARP-dependent mA RNA methylation at DNA lesions is subsequently accompanied by active DNA demethylation. RNA Biol 19:1153–1171 36382943 10.1080/15476286.2022.2139109 PMC9673957

[CR32] Lippincott-Schwartz J, Snapp EL, Phair RD (2018) The development and enhancement of FRAP as a key tool for investigating protein dynamics. Biophys J 115:1146–1155 30219286 10.1016/j.bpj.2018.08.007 PMC6170817

[CR33] Mlynsky V, Janecek M, Kührová P, Fröhlking T, Otyepka M, Bussi G, Banás P, Sponer J (2022) Toward convergence in folding simulations of RNA tetraloops: comparison of enhanced sampling techniques and effects of force field modifications. J Chem Theory Comput 18:2642–2656 35363478 10.1021/acs.jctc.1c01222

[CR34] Monteith JL, Unsworth MH (2013) Principles of environmental physics, plants, animals, and the atmosphere, 4th edn. Academic Press, London, p 395

[CR35] Nermut MV, Drasil V (1958) Changes in the dry weight and the deoxyribonucleic acid content of *Proteus-vulgaris* caused by penicillin. Nature 181:1740–1742 13566132 10.1038/1811740b0

[CR36] Palecek E (1960) Oscillographic polarography of highly polymerized deoxyribonucleic acid. Nature 188:656–657 13732209 10.1038/188656a0

[CR37] Palecek E, Bartosík M (2012) Electrochemistry of nucleic acids. Chem Rev 112:3427–3481 22372839 10.1021/cr200303p

[CR38] Palecek E, Tkác J, Bartosík M, Bertók T, Ostatná V, Palecek J (2015) Electrochemistry of nonconjugated proteins and glycoproteins. Toward sensors for biomedicine and glycomics. Chem Rev 115:2045–2108 25659975 10.1021/cr500279h PMC4360380

[CR39] Rabi II (1937) Space quantization in a gyrating magnetic field. Phys Rev 51:0652–0654

[CR40] Ripin N, Boudet J, Duszczyk MM, Hinniger A, Faller M, Krepl M, Gadi A, Schneider RJ, Sponer J, Meisner-Kober NC, Allain FHT (2019) Molecular basis for AU-rich element recognition and dimerization by the HuR C-terminal RRM. P Natl Acad Sci USA 116:2935–2944 10.1073/pnas.1808696116 PMC6386705 30718402

[CR41] Röntgen WC (1895) Ueber eine neue Art von Strahlen. Vorläufige Mitteilung Aus den Sitzungsberichten der Würzburger Physik-medic. Gesellschaft, Würzburg, pp 137–147

[CR42] Skalka M, Matyasova J, Cejkova M (1976) DNA in chromatin of irradiated lymphoid-tissues degrades invivo into regular fragments. FEBS Lett 72:271–274 16386038 10.1016/0014-5793(76)80984-2

[CR43] Slabáková E, Culig Z, Remšík J, Souček K (2017) Alternative mechanisms of miR-34a regulation in cancer. Cell Death Dis 8:e3100 29022903 10.1038/cddis.2017.495 PMC5682661

[CR44] Šlotová J, Karpfel Z, Kubíčková D (1971) Contribution to the study on the reparative effect of exogenous DNA in the irradiated meristem of *Vicia faba*. Biol Plant 13:69–78

[CR45] Soska J, Drasil V, Karpfel Z (1958) The transplantation of blood-forming tissue in irradiated animals. Transplan B 5:68–68

[CR46] Sponer J, Leszczynski J, Hobza P (1996) Nature of nucleic acid-base stacking: nonempirical ab initio and empirical potential characterization of 10 stacked base dimers. Comparison of stacked and H-bonded base pairs. J Phys Chem-Us 100:5590–5596

[CR47] Stadlbauer P, Krepl M, Cheatham TE, Koca J, Sponer J (2013) Structural dynamics of possible late-stage intermediates in folding of quadruplex DNA studied by molecular simulations. Nucleic Acids Res 41:7128–7143 23700306 10.1093/nar/gkt412 PMC3737530

[CR48] Stixová L, Bártová E, Matula P, Danek O, Legartová S, Kozubek S (2011) Heterogeneity in the kinetics of nuclear proteins and trajectories of substructures associated with heterochromatin. Epigenet Chromatin 4(1):5 10.1186/1756-8935-4-5 PMC3068931 21418567

[CR49] Stros M (2010) HMGB proteins: interactions with DNA and chromatin. BBA-Gene Regul Mech 1799:101–113 10.1016/j.bbagrm.2009.09.008 20123072

[CR50] Vorlickova M, Kypr J, Kleinwachter V, Palecek E (1980) Salt-induced conformational-changes of poly(Da-Dt). Nucleic Acids Res 8:3965–3973 7443518 10.1093/nar/8.17.3965 PMC324207

[CR51] Watson JD, Crick FHC (1953) Molecular structure of nucleic acids: a structure for deoxyribose nucleic acid. Nature 171:737–738 13054692 10.1038/171737a0

[CR52] Zgarbová M, Otyepka M, Sponer J, Mládek A, Banás P, Cheatham TE, Jurecka P (2011) Refinement of the Cornell et al. nucleic acids force field based on reference quantum chemical calculations of glycosidic torsion profiles. J Chem Theory Comput 7:2886–2902 21921995 10.1021/ct200162x PMC3171997

